# Advances in studying whole mouse brain vasculature using high-resolution 3D light microscopy imaging

**DOI:** 10.1117/1.NPh.9.2.021902

**Published:** 2022-04-05

**Authors:** Hannah C. Bennett, Yongsoo Kim

**Affiliations:** The Pennsylvania State University, Department of Neural and Behavioral Sciences, Hershey, Pennsylvania, United States

**Keywords:** cerebrovasculature, three-dimensional light microscopy, block-face imaging, light sheet fluorescence microscopy, neurovascular unit, brain

## Abstract

**Significance:**

The cerebrovasculature has become increasingly recognized as a major player in overall brain health and many brain disorders. Although there have been several landmark studies to understand details of these crucially important structures in an anatomically defined area, brain-wide examination of the whole cerebrovasculature, including microvessels, has been challenging. However, emerging techniques, including tissue processing and three-dimensional (3D) microscopy imaging, enable neuroscientists to examine the total vasculature in the entire mouse brain.

**Aim:**

Here, we aim to highlight advances in these high-resolution 3D mapping methods including block-face imaging and light sheet fluorescent microscopy.

**Approach:**

We summarize latest mapping tools to understand detailed anatomical arrangement of the cerebrovascular network and the organizing principles of the neurovascular unit (NVU) as a whole.

**Results:**

We discuss biological insights gained from studies using these imaging methods and how these tools can be used to advance our understanding of the cerebrovascular network and related cell types in the entire brain.

**Conclusions:**

This review article will help to understand recent advance in high-resolution NVU mapping in mice and provide perspective on future studies.

## 1 Introduction

The cerebrovasculature serves as a key infrastructure that is necessary to sustain the most energy-demanding organ in the body, the brain.^1^ Blood supply in the brain is actively regulated based on local energy consumption, largely by neurons, via interaction between perivascular cells, astrocytes, and vasomotor neurons, broadly termed as the neurovascular unit (NVU).^2^^–^^4^ The vasculature also plays essential role to prevent the accumulation of toxic waste.^5^ For example, large-scale vasodilation during sleep is important for the clearance of the brain’s metabolic waste.^6^ Not surprisingly, cerebrovascular dysfunction has been heavily implicated in many neurological (e.g., stroke), neurodegenerative (e.g., Alzheimer’s), and even neurodevelopmental disorders (e.g., autism).^7^^,^^8^ Thus, it is critically important to understand how the entire cerebrovascular network is structurally organized and how it changes during pathological conditions.

Given the complexity of this system, a high-resolution analysis of the cerebrovasculature is needed to understand the cellular organization of the NVU across multiple brain regions. This type of approach is not feasible using current *in vivo* methods, such as MRI and functional ultrasound, due to the lack of resolution necessary to visualize the vast network of microvessels extending beyond the major arterial branches of the circle of Willis.^9^^–^^11^ Additionally, *in vivo* two-photon imaging and *in vivo* optical coherence tomography have limited imaging depth.^12^^,^^13^ Although immunohistochemical and electron microscopy studies have also revealed crucial insights into the NVU major players, these modalities are often limited to localized brain regions, as they are not easily applied to whole-brain studies. However, several studies suggest that there are major differences in cellular composition, energy demand, as well as a diverse array of functions among different regions of the isocortex, let alone in the rest of the brain.^14^^,^^15^ This indicates that the vascular characteristics and organization of one brain region may not be applicable to others. As such, there is a need for network level and brain-wide study of the cerebrovascular organization and regional heterogeneity in the brain, to better understand their crucial functions, as well as any potential vulnerabilities upon pathological conditions.

Fortunately, technological innovation has paved the way for studies of cerebrovascular mapping in the whole mouse brain. Although the list of methods continues to grow, the focus here will include a current adaptation of several cellular resolution *ex vivo* imaging methods that can be loosely categorized into block-face imaging with serial sectioning and light sheet fluorescence microscopy (LSFM). We will also discuss the sample processing and vessel labeling strategies that contribute to an integral part of the imaging process. These imaging modalities provide the means to study details of the cerebrovasculature, and each has its own set of strengths and limitations. Importantly, these studies are technically challenging, not only in terms of imaging, but also in terms of analysis pipelines that require high-level computational skill. Given this, as well as the rapid expansion of these modalities and our understanding regarding the importance of the brain vasculature, it has become necessary to synthesize the knowledge and resources gained from recent research efforts.

**Fig. 1:**
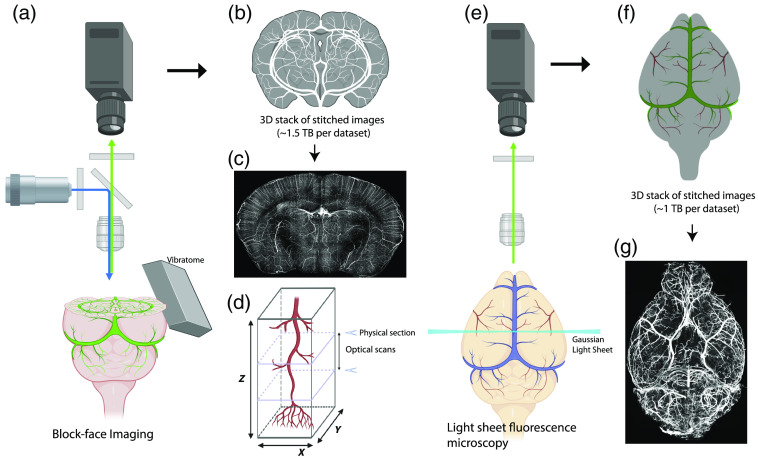
Overview of block-face imaging versus LSFM. (a–d) STPT is used as an example of the block-face image. (a) STPT uses a built-in vibratome to physically remove brain slices after block-face imaging. (b) Results produce 3D stack of 2D high-resolution images with 1 to 2 TB in size for the whole mouse brain. (c) Maximum intensity projection (MIP) of coronal sections (1000-μm thick) with fluorescein isothiocyanate (FITC) filled vascular signals. (d) Physical serial section with optical scans in between physical sectioning can provide sufficient high resolution in 3D to resolve the entire cerebrovasculature. (e) LSFM to visualize the entire cerebrovasculature from an intact and cleared mouse brain. (f) LSFM produces about 1 to 2 TB data to visualize the whole mouse brain vasculature. (g) MIP of horizontal sections (6600-μm thick) with artery-specific antibody labeling.

## 2 Block-Face Imaging Modalities Used to Study the Brain Vasculature

Several serial sectioning-based methods for mesoscale whole-brain mapping have been developed and extensively utilized for neuronal cell type mapping.^14^^,^^16^^,^^17^ These variations utilize tissue sectioning techniques paired with light microscopy to overcome difficulties associated with the imaging depth limitations and to achieve high-resolution images of the entire brain in one imaging session.^18^^,^^19^ These methods have been recently adapted for studying the cerebrovasculature, due to the ability to obtain images at micron and submicron resolution, which is critical for imaging and tracing the capillaries [Figs. 1(a)–1(d)].^9^^,^^10^^,^^20^

There are several variations of block-face imaging techniques including, micro-optical sectioning tomography (MOST), knife-edge scanning microscopy (KESM), serial two-photon tomography (STPT), and block-face serial microscopy tomography (FAST). MOST and KESM are two variations of similar technologies that consist of a microtome paired with a light microscope.^9^^,^^19^^,^^21^^–^^24^ Due to the incorporation of the microtome and hardened samples, these techniques achieve resolution at $∼0.35\times 0.35\times 1\;{\mu m}^{3}$ (x,y,z), with some variation in the z step size.^9^^,^^23^ Other recognized methods for vascular whole-brain mapping are STPT and FAST.^14^^,^^18^ STPT harnesses the cellular resolution capabilities of two-photon imaging and pairs this with the applications of vibratome sectioning.^18^ STPT imaging with optical sectioning can achieve cellular resolution typically ranges from $0.3\times 0.3\times 1\;\mu m^{3}$ to $1\times 1\times 5\;\mu m^{3}$ (x,y,z) resolution.^10^^,^^25^ Moreover, FAST is a similar technology that utilizes a spinning disk-based confocal microscope that is paired with a microslicer, resulting in a resolution of $0.7\times 0.7\times 5\;\mu m^{3}$ (x,y,z).^26^ Importantly, this method is faster than STPT, as an adult mouse brain can be imaged in <3  h.^26^

### 2.1 Labeling Strategies for Block-Face Microscopy Methods

To study the vasculature at cellular resolution, several labeling methods for block-face microscopy have been developed. Some of the first vasculature-focused studies, using both MOST and KESM, were paired with the gelatin-Indian Ink perfusion method.^27^^,^^28^ Recently, several studies have also incorporated modified Nissl staining, which provides both vascular and cytoarchitectural information within the same sample.^9^^,^^23^ Alternatively, STPT has been used consistently to map the neuronal cell types using transgenic animals expressing fluorescent proteins in the cells of interest. Recently, this technique has been adapted for studying cerebrovasculature.^10^^,^^20^^,^^25^^,^^29^ For this purpose, transgenic animals expressing endothelial markers are possible, such as the Tie2-RFP transgenic mouse model.^22^ More commonly, perfusion-based methods with FITC-tagged albumin gel mixtures have been widely used for complete vessel lumen labeling.^10^^,^^20^^,^^25^^,^^30^ The benefit of FITC-fill and other perfusion-based methods is that it can be adapted for any animal model of interest, particularly disease models, without the need for transgenic reporter animals.^31^ Importantly, comparisons between *in vivo* two-photon imaging and *ex vivo* imaging in the same animal showed that perfusion-based vessel labeling preserves the overall geometry of cerebrovasculature (e.g., radii and shape).^10^^,^^25^ Finally, FAST imaging studies have also utilized fluorescently tagged lectin labeling of the vasculature, which involves the incorporation of dye-based labeling rather than perfusion-based vessel filling.^26^

### 2.2 Drawbacks to Block-Face Methods

However, there are drawbacks for perfusion-based labeling and sectioning-based imaging methods for studying the vasculature. For example, the FITC-fill and Indian-ink perfusion techniques can result in incomplete filling of the vessel lumen, which is also the case for other methods that rely on vessel filling.^10^^,^^20^^,^^25^^,^^30^ This can lead to the loss of precious data and add uncertainty regarding whether poorly vascularized regions are biological or due to labeling procedural issues. Another issue with both perfusion and dye-based labeling methods is technical limitations in distinguishing vascular compartments (e.g., arteriole versus venule) and identifying the directionality of blood flow. Importantly, methods such as MOST, KESM, STPT, and FAST all require tissue sectioning, so any unstable cutting may lead to arbitrary vessel breakage at the locations of physical sectioning of the tissue.^10^^,^^25^ Moreover, sample preparation time for many studies using MOST can take several weeks of sample processing steps followed by ∼1  week of imaging, which is no small time investment.^9^ Similarly, vascular imaging using STPT can take quite a while, as one study reported that full imaging of an adult mouse brain takes ∼5  days to complete.^25^ On the other hand, STPT imaging does not require any lengthy sample processing prior to imaging.

## 3 Light Sheet Fluorescence Microscopy Imaging for Vascular Studies

Recently, the optimization of brain clearing methods has paved the way for use of LSFM to examine the cerebrovasculature in a whole mouse brain. LSFM consists of a light microscope, in which the cleared sample is illuminated with a two-dimensional (2D) plane of thin light with a detector positioned at an orthogonal angle [Figs. 1(e)–1(g)].^11^^,^^32^^,^^33^ Although, LSFM is referred to as one major imaging category, there are several different types of light sheet microscope configurations. For example, types of LSFM can include selective plane illumination microscopy (SPIM) as well as inverted, multiview, Bessel beam, and stimulation emission depletion variations of SPIM.^34^ The various configurations of LSFM primarily differ in the positioning of the sample, objective, and light beams to optimize light sheet penetration while also preventing the introduction of imaging or sample-related artifacts.^34^ Most studies of the vasculature using LSFM have primarily utilized the standard Gaussian form of SPIM.^11^^,^^32^^,^^34^ However, the strengths of Bessel beam SPIM were recently demonstrated for capturing the high-fidelity imaging of the brain vasculature while avoiding the streaking artifacts that result from Gaussian illumination.^35^ Importantly, LSFM resolution can vary widely depending on the microscope and objective lens used, but overall this method can achieve submicron cellular resolution imaging for whole three-dimensional (3D) volumes.^11^^,^^33^ A major strength of this imaging modality is that optical sectioning achieved through LSFM prevents the need for physical tissue sectioning, which helps to avoid the potential artifact of vessel breakage caused by physical cutting.^19^^,^^29^ Moreover, applications of vessel labeling strategies avoid the need for transgenic animals and allow for the opportunity to examine the vasculature of multiple species, including human tissue.^36^ Finally, the sheer speed of data acquisition for LSFM can result in terabytes of whole-brain datasets captured in only a few hours per brain.^11^^,^^33^

### 3.1 Labeling Strategies for LSFM Vascular Mapping

To conduct LSFM for vascular imaging, tissue samples must be optically cleared with optional immunolabeling. Several clearing methods have been developed and adapted for studying various animal models and cellular markers. A commonly used clearing method is iDISCO, a variation of solvent-based clearing, which is useful for vascular labeling but is associated with a slight volumetric shrinkage of the tissue and quenching of endogenous fluorescence.^33^^,^^37^^,^^38^ A modified method, called fDISCO, improved the preservation of these endogenous fluorescent markers.^39^ Aqueous-based methods, such as SHIELD, are particularly useful for an array of markers, while also improving retention of endogenous fluorescence and shortening tissue processing time.^40^^–^^42^ However, aqueous-based methods do have caveats associated with tissue swelling.^40^^–^^42^ Finally, the CLARITY method for brain clearing has utilized vessel labeling through lectin and claudin-5, which improved the depth of antibody penetration and speed of sample processing compared with iDISCO.^40^^,^^43^^,^^44^ Although there are a variety of brain clearing methods, these techniques can all serve for whole-brain imaging using LSFM.^32^^,^^38^^,^^45^^,^^46^

In fact, two recent labeling methods highlighted the capabilities of whole-brain immunolabeling by capturing each compartment of the vessel tree within the same sample. One study utilized modified iDISCO clearing, followed by a combination of antibodies to uniquely label arteries, veins, and capillaries with three different colors.^33^ The authors sought to improve vessel staining throughout an entire mouse brain hemisphere by combining primary antibodies produced from the same source species.^33^ This clever strategy helped to achieve strong labeling for different vascular compartments, including the distribution of arterioles and venules across all brain regions, thereby providing directionality to the vascular network.^33^ Moreover, this study also demonstrated that the autofluorescence of blood could be used as a vascular label for LSFM.^33^ Another study by Todorov et al.,^11^ utilized wheat germ agglutinin which binds the glycocalyx of the endothelial lining of blood vessels, paired with Evans Blue dye to label large vessels based on its affinity for albumin in combination with 3DISCO-based brain clearing. These method-focused studies have paved the way for in-depth interrogation of the vascular network.

### 3.2 Drawbacks to Brain Clearing and LSFM-Based Methods

As with other modalities, brain clearing methods and LSFM do have disadvantages. One major issue is the volumetric swelling or shrinkage associated with most brain clearing methods, which significantly impacts volumetric and vessel parameter analysis.^10^^,^^11^^,^^33^^,^^37^ Comparison of the cerebrovascular analysis showed substantial discrepancies in vascular densities amongst studies using STPT, LSFM, and MOST methods.^10^ Potential volume distortion by different clearing or related sample preparation methods can partially explain a large difference in reported regional vascular densities, between STPT and LSFM. Thus, vascular analysis with different tissue clearing and LSFM needs to account for such potential procedural artifacts by comparing its results with data from minimal distortion (e.g., MRI or *in vivo* two-photon imaging) and accordingly adjusting measurements. Additionally, while LSFM imaging itself is rather quick (a few hours per mouse brain), the sample processing time required for most brain clearing methods takes several weeks.^33^^,^^37^^,^^39^^,^^40^ Finally, another caveat associated with immunolabeling is that the staining is only as good as the antibody or dye that is used. Moreover, the extraordinary size of the data provided by imaging with higher magnification objective lenses confer issues with data storage and further complexities for analysis.

## 4 Analytical Steps for High-Resolution Cerebrovascular Analysis

To properly analyze the sheer amount of data produced by 3D vascular imaging, there are several computational steps. For example, algorithms and machine learning pipelines are required to perform 3D image processing (e.g., stitching), segmentation, binarization, skeletonization, tracing, and finally registration to a reference brain atlas, all of which have been major technical hurdles in furthering understanding of the vasculature in different brain regions.^11^^,^^33^ Fortunately, several studies have generated image processing and analysis pipelines for both categories of imaging discussed here. A prime example is STPT, for which two groups released their pipelines built according to FITC-based vessel filling, as well as useful visualization tools for the analyzed data.^10^^,^^25^ Similar analysis tools have been developed for LSFM, using different labeling approaches, to provide solutions for examining different vascular compartments.^11^^,^^33^ These open-source analysis tools will become instrumental for network-level studies of these structures and detailed quantitative investigations of the region- and disease-specific studies.

## 5 Insight Gained from High-Resolution Cerebrovascular Imaging in the Whole Brain

**Fig. 2:**
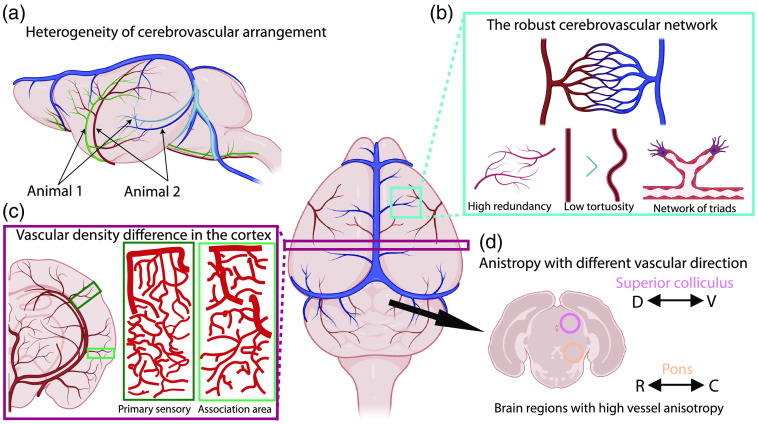
Summary of cerebrovascular network organization in the mouse brain. (a) The spatial arrangement of cerebrovasculature is variable across different animals. (b) Robustness of the cerebrovascular network. Cerebrovasculature from young mouse brains showed high redundancy and relatively straight vasculature with triad branching features. (c) The primary sensory cortices showed higher vascular length density than association cortices. (d) Brain stem areas show a high degree of anisotropy, with preferential directionality in a vascular arrangement. D, dorsal; V, ventral; R, rostral; C, caudal.

These various imaging technologies have provided the means to study the macro- and microvasculature at cellular resolution and uncover the anatomy, organization, geometry, and intricate characteristics of this complex network. One major step forward involved the 3D reconstructed rendering of pial surface vessels and annotation of the first whole-brain vascular atlas with stereotaxic coordinates at 1-μm voxel resolution.^9^ The authors also provided detailed annotations of the venous system, which had previously been underreported at this level of detail.^9^ As one might expect, the diameter of first- and second-order arteries and veins were reported to be similar between left and right hemispheres and across different mouse brains.^9^ However, another recent study demonstrated that large surface vessels vary considerably in anatomical position as well as the locations of branch points, across different animals and even between hemispheres within the same animal.^25^ This surprising result, depicted in Fig. 2(a), suggests that vascular development and organization may not be as stereotypical as previously thought.

The real power of high-resolution 3D vascular mapping is its ability to image and resolve the entire network of capillaries in the brain. Microvascular networks in the adult mouse brain prevent rarefaction through redundancy and low tortuosity.^10^ Additionally, various analyses uncovered that vessel branches were commonly triadic and overall vessels tended to be relatively straight.^10^ As expected, branching density was correlated with microvessel length density.^33^ Although these findings are not particularly unexpected, they do demonstrate how the vasculature is able to support the energy-expensive functions of the brain both efficiently and effectively, under normal conditions. Due to the aforementioned robust nature of this network as illustrated in Fig. 2(b), it was determined that the vasculature system would fail if over 40% of the capillaries, defined as vessels with a radius of <3.5  μm, were to be lost or become occluded.^10^ Considering the well-documented loss of capillaries in normal and pathological aging [e.g., Alzheimer’s disease (AD)], it will be interesting to measure how the loss of microvessels can negatively affect regional energy supply linked with neuronal health.^36^^,^^47^^,^^48^

Recent studies also highlight regional differences in cerebrovascular organization.^10^^,^^25^^,^^33^ For instance, the cortex is composed of primary motor-sensory and higher association areas, each with distinct neuronal subtype compositions and anatomical connectivity.^14^^,^^49^ We recently identified that primary motor-sensory regions, such as the somatosensory cortex, were found to have higher vascular length density than association regions, such as the prefrontal cortex, as shown in Fig. 2(c).^25^ Importantly, a similar pattern was observed in the thalamus and the dorsal striatum, which are heavily connected with these primary motor-sensory and association cortices, thus providing conserved vascular density patterns across cortical and subcortical networks.^25^^,^^50^^,^^51^ Moreover, even aside from density, the directional organization of the vasculature differs between these anatomical regions. For instance, vasculature in the isocortex is more preferably organized in radial (dorso-ventral) orientation rather than planar (medial-lateral or rostro-caudal) directions.^33^ Interestingly, overall vascular direction (largely influenced by the abundance of microvessels) in the deep layers of primary sensory areas shows the dominant anterior-posterior direction for interareal vascular connections,^25^ thereby providing additional evidence for vascular regional heterogeneity in the normal adult mouse brain. The vascular organization of subcortical brain regions is also becoming increasingly recognized for unique differences with other brain areas. For example, there is regionally distinct anisotropy that is particularly strong in the pons and superior colliculus [Fig. 2(d)].^10^ Moreover, vasculature in white matter tracts have reduced vascular density compared with cortical areas, as well as increased vessel length with reduced branching density.^10^^,^^33^ These findings further suggest that subcortical brain areas may contain distinctly different vascular organization from what is seen in the cortex. Even aside from regional heterogeneity within the brain, it is also important to account for differences amongst various animal models that are often used to represent the normal condition. For example, Todorov et al.,^11^ examined vascular differences across C57B/l6, CD1, and BALB/c lines and found that CD1 mice showed increased connections between major artery branches compared with the other mouse strains. Although more related to study design, this does provide rather important considerations when choosing animal models, especially for studies using disease models with mixed background strains.

High-resolution cerebrovascular datasets provide crucial structural information to simulate blood flow using in silico modeling.^52^^,^^53^ Sample collection (e.g., perfusion and fixation) and labeling procedures (e.g., vessel filling with dyes or 3D immunolabeling with tissue clearing) may introduce distortion. Thus, careful validation is needed to confirm that vascular geometry measurements (e.g., vascular diameter, shape, connectivity) from fixed brains are matched or correlated with *in vivo* conditions.

## 6 High-Resolution 3D Vascular Mapping in Pathological Animal Models

Dysfunction in the cerebrovasculature has been highly implicated in many brain disorders including cerebral ischemia and neurodegenerative diseases.^2^^,^^48^^,^^54^ High-resolution 3D vascular mapping provides an unprecedented opportunity to quantitively examine loss and rearrangement of cerebrovasculature, including microvessels, upon pathological conditions. A previous study combined the use of FITC-conjugated albumin gel infusion with 3DISCO solvent-based clearing and LSFM to study the impact of cerebral ischemia on the vasculature of the whole brain.^55^ As expected, middle cerebral artery occlusion (MCAO) led to a loss of vessel labeling in the striatum, with further analysis showing a selective loss of capillaries in the overall 64% loss of vessel density in this region.^55^ Moreover, MCAO led to vessel damage such as tortuous loops or “candelabrum-like” structures, as well as a disconnected beaded-like appearance in regions of the infarcted tissue.^55^ Yet another study of MCAO demonstrated that while capillary branch density in the infarcted tissue of the somatosensory cortex was not significantly different, capillaries showed reorganization to orient themselves towards the ischemic center of the infarct.^33^ Although stroke is a clear avenue for the pathological study of the brain vasculature, these results indicate that vascular compensation and repair may provide an intriguing insight into how the vascular network functions and respond to various insults.

Recently, there is increased interest in cerebrovascular development and its roles in developmental disorders, such as autism spectrum disorders.^8^ One such study looked at how changes in sensory input and therefore neuronal activity could impact the vasculature during brain development.^33^ Given the high density of vasculature in the auditory cortex, the authors studied a model of congenital deafness to investigate how vessel density would respond to a reduction in neuronal activity in this region.^33^ Interestingly, vessel branch point density was only slightly decreased in the auditory cortex, while there was an increase in other sensory areas such as the vibrissa and visual cortices and even some association cortices.^33^ These results demonstrate how intricately related these systems are, while further indicating that the vasculature may also contribute to neurodevelopmental disorders.

Finally, mounting evidence suggests these important structures may also play a major role in neurodegenerative diseases. For instance, recent evidence suggests that AD is heavily influenced by vascular impairment.^22^^,^^31^ For example, a MOST imaging study identified that the dentate gyrus within the hippocampus of a mouse model of AD undergoes a significant reduction in vascular diameter and density, as well as irregular morphological changes.^22^ Even when studied in a regionally specific manner, vascular changes were evident in this model. Similarly, an STPT imaging study of the transgenic APP/PS1 model of AD identified that aging conferred a decrease in vascular density.^31^ Recently, brain clearing methods were used comprehensively 3D label amyloid plaques in an amyloid β accumulation mouse model of AD.^36^ This study highlighted the strengths of whole-brain immunolabeling and LSFM by showing labeling of amyloid plaques within a 3D context while also including the vasculature and glial cells, in both an AD mouse model and in human AD brain tissue.^36^

Together, these studies demonstrate how the power of high-resolution 3D mapping methods reveals the crucial roles of the brain vasculature throughout the lifespan and in various pathologies.

## 7 Examination of Cerebrovasculature in the Context of NVU Cell Types

3D mapping methods also provide means to examine how cerebrovasculature is structurally organized in the context of other NVU cell types including neurons, glia, and perivascular cells. Neurons expressing neuronal nitric oxide synthase (nNOS) are particularly important for increasing blood flow upon activation to mediate neurovascular coupling.^56^^–^^60^ However, neuronal activity is not the only factor that controls vascular changes. Astrocytes densely cover the cerebrovasculature with their end-feet, which help to maintain vascular integrity and permeability, and regulate vasomotility for brain energy homeostasis.^61^^–^^64^ Additionally, pericytes, a mural cell type that encapsulates the microvasculature, also contribute to the control of blood flow at the level of the capillaries.^65^^–^^68^ Recently, we demonstrated the capabilities of 3D mapping through the generation of the first whole-brain mapping resources for both nNOS neurons and pericytes using STPT.^25^ Through these cell type maps, we revealed that nNOS neuron distribution in the isocortex negatively correlated with vascular density, while pericyte coverage positively correlated with the vasculature length density.^25^ Further interrogation also identified that vascular density shows particularly strong correlations with energy-demanding parvalbumin interneurons in the isocortex.^25^ These findings provide evidence of the dynamic partnership with neuronal cell types and spurs intriguing questions regarding how vascular and neuronal development is orchestrated. Moreover, brain clearing and immunolabeling methods provide the opportunity to study the relationships between cell types and the vasculature within the same brain. For example, a recent iDISCO based study highlighted the importance of studying cerebral amyloid angiopathy in the context of the vasculature and glial cell types, such as astrocytes and microglia.^36^ Clearly, integration of cellular relationships with the vasculature will help to provide insight into how the entire system of the brain is structurally organized and how it responds to injury and disease.

### 7.1 Putting This All Together

Each of these imaging modalities presents with their own strengths and problems and often provide complementary findings that further improve this field of study. Importantly, these methods also allow for the study of hard-to-reach subcortical structures at micron and submicron resolution, which have been largely understudied. For example, in only a few years these studies have identified striking differences between cortical and subcortical regions of the brain, beginning to unveil the detailed structure and organizing principles of the cerebrovasculature. These findings, some of which are summarized in Fig. 2, will now provide the basis for future studies of the complex and intricate nature of the vasculature. Moreover, the investment in analysis pipelines for multiple imaging modalities will certainly improve access to various research groups that may not otherwise be able to handle the advanced computational expertise necessary to analyze this particularly complex data. Additionally, solving the problem of analyzing vascular data, which is arguably one of the more complex structures in the body, will inform the quantitative study of associated NVU components, from a population perspective. Current evidence also raises questions regarding vascular development, in terms of whether cerebrovascular development is stereotypical and how changes in vascular growth could have repercussions in terms of the progression of neurodevelopmental disorders. In the same vein, the use of these technologies to compare animal models to human tissue will become increasingly important to distinguish species-specific vascular characteristics and organizational differences, particularly between rodent models and humans. This will become increasingly important when considering how disease processes may actually differ between human tissue and preclinical models, which will have significant implications for the testing of potential treatments. Although the sheer size of the human brain presents enormous technical challenges in sample preparation (e.g., tissue clearing), imaging of the whole 3D volume, and related data size (estimated in petabyte-scale), the rapid advance in tissue engineering, imaging methods, computer hardware, and analytical tools begins to open new possibilities to examine detailed cellular detailed in the intact human brain.^46^^,^^69^^,^^70^ It is becoming clear that there is an exciting future for systemic neuroscience when it considers how supportive cell types, such as vasculature, play an instrumental role in the maintenance of brain health and the development of disease.
